# Etiologic features of diarrheagenic microbes in stool specimens from patients with acute diarrhea in Thailand

**DOI:** 10.1038/s41598-020-60711-1

**Published:** 2020-03-04

**Authors:** Kazuhisa Okada, Warawan Wongboot, Watcharaporn Kamjumphol, Namfon Suebwongsa, Piyada Wangroongsarb, Pipat Kluabwang, Nuttagarn Chuenchom, Witaya Swaddiwudhipong, Thanee Wongchai, Weerawat Manosuthi, Norrathep Assawapatchara, Patchanee Khum-on, Patpong Udompat, Chareeya Thanee, Suwatthiya Kitsaran, Lakkana Jirapong, Charoen Jaiwong, Supalert Nedsuwan, Chotipong Siripipattanamongkol, Pilailuk Akkapaiboon Okada, Siriporn Chantaroj, Sho Komukai, Shigeyuki Hamada

**Affiliations:** 1 Thailand-Japan Research Collaboration Center on Emerging and Re-emerging Infections, Nonthaburi, Thailand; 2 Research Institute for Microbial Diseases, Osaka University, Osaka, Japan; 3 National Institute of Health, Department of Medical Sciences, Nonthaburi, Thailand; 4 Department of Pediatrics, Maesot General Hospital, Tak, Thailand; 5 Department of Medicine, Maesot General Hospital, Tak, Thailand; 6 Department of Community and Social Medicine, Maesot General Hospital, Tak, Thailand; 7 Department of Clinical Laboratory, Maesot General Hospital, Tak, Thailand; 8 Department of Medicine, Bamrasnaradura Infectious Diseases Institute, Nonthaburi, Thailand; 9 Department of Medicine, Ranong Hospital, Ranong, Thailand; 10 Department of Medical Technology, Chum Phae Hospital, Khon Kaen, Thailand; 11 Department of Community and Social Medicine, Prapokklao Hospital, Chanthaburi, Thailand; 12 Department of Pediatrics, Sunpasitthiprasong Hospital, Ubon Ratchathani, Thailand; 13 Department of Medicine, Sunpasitthiprasong Hospital, Ubon Ratchathani, Thailand; 14 Department of Radiology, Samutsakhon Hospital, Samutsakhon, Thailand; 15 Department of Pediatrics, Chiangrai Prachanukroh Hospital, Chiang Rai, Thailand; 16 Department of Preventive and Social Medicine, Chiangrai Prachanukroh Hospital, Chiang Rai, Thailand; 17 Department of Medicine, Chiangrai Prachanukroh Hospital, Chiang Rai, Thailand; 18 Department of Integrated Medicine of Graduate School of Medicine, Osaka University, Osaka, Japan

**Keywords:** Infectious-disease epidemiology, Clinical microbiology, Infectious-disease diagnostics, Viral epidemiology, Diarrhoea

## Abstract

Many microbial species have been recognized as enteropathogens for humans. Here, we predicted the causative agents of acute diarrhea using data from multiplex quantitative PCR (qPCR) assays targeting 19 enteropathogens. For this, a case-control study was conducted at eight hospitals in Thailand. Stool samples and clinical data were collected from 370 hospitalized patients with acute diarrhea and 370 non-diarrheal controls. Multiple enteropathogens were detected in 75.7% and 13.0% of diarrheal stool samples using multiplex qPCR and bacterial culture methods, respectively. Asymptomatic carriers of enteropathogens were found among 87.8% and 45.7% of individuals by qPCR and culture methods, respectively. These results suggested the complexity of identifying causative agents of diarrhea. An analysis using the quantification cut-off values for clinical relevance drastically reduced pathogen-positive stool samples in control subjects from 87.8% to 0.5%, whereas 48.9% of the diarrheal stool samples were positive for any of the 11 pathogens. Among others, rotavirus, norovirus GII, *Shigella*/EIEC, and *Campylobacter* were strongly associated with acute diarrhea (*P*-value < 0.001). Characteristic clinical symptoms, epidemic periods, and age-related susceptibility to infection were observed for some enteropathogens. Investigations based on qPCR approaches covering a broad array of enteropathogens might thus improve our understanding of diarrheal disease etiology and epidemiological trends.

## Introduction

Diarrheal diseases are one of the major causes of mortality and morbidity worldwide, especially during the first 5 years of life for individuals subjected to malnutrition^1–3^. Diarrhea can be defined by increased stool frequency, liquidity, or volume^4^. A wide range of enteropathogens including bacteria, viruses, and protozoa have been recognized as the causative agents of infectious diarrhea^5,6^. Several enteropathogens act either directly by modulating epithelial ion transport systems and barrier functions or indirectly via neuropeptide secretion, induction of inflammation, or by compromising intestinal absorption^7^. Thus, the timely identification of causes of acute diarrhea can lead to appropriate treatment, prevention, and control measures. Molecular assays targeting nucleic acid markers facilitate the screening of a broad range of enteropathogens in stool samples, consuming much less time than conventional methods such as cell culture, microscopy, and antigen-based tests. However, due to the high sensitivity of these nucleic acid amplification assays, high rates of asymptomatic carriers and mixed infection cases are commonly reported, particularly in developing country settings^8–12^. Several studies have shown correlations between pathogen load and severity, or have accurately diagnosed individual pathogens using qPCR assays with optimal cut-off values^13–21^.

In this study, we predicted the etiological agents of acute diarrhea using stool samples from hospitalized patients in Thailand using our quantitative pathogen detection procedure^22^ with clinically relevant cut-off values. We further investigated characteristic clinical symptoms, epidemic periods, and age-related susceptibility to infection associated with the detected enteropathogens.

## Results

### Detection of diarrheagenic microbes in stool specimens using multiplex qPCR assays

**Figure 1:**
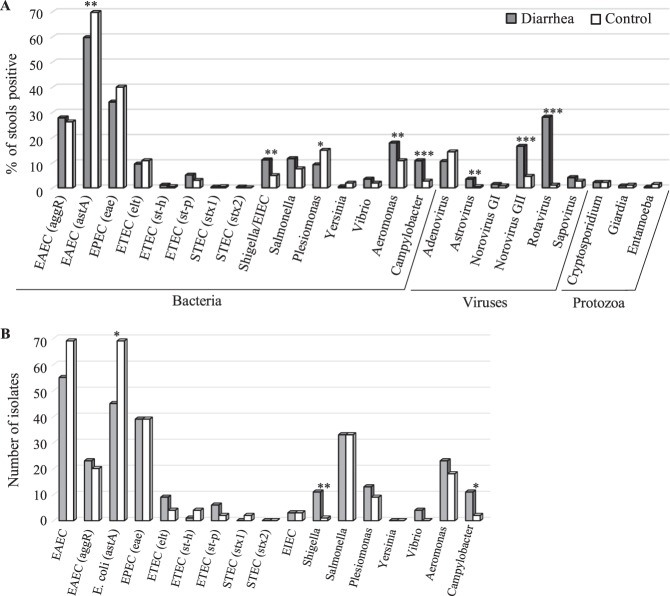
Detection of enteropathogens in 370 diarrheal and 370 non-diarrheal control subjects. **(A**) Proportions of patients and control subjects who tested positive by qPCR assays. (**B**) Number of isolates by bacterial culture methods. *P < 0.05; **P < 0.01; ***P < 0.001.

**Figure 2:**
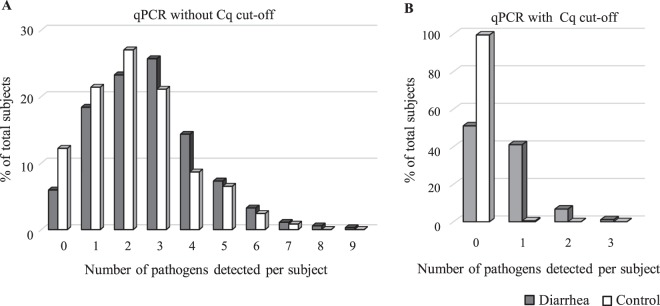
Number of pathogens detected per subject using qPCR among 370 each of diarrheal (an average of 2.7 ± 0.1 (standard error) pathogens) and non-diarrheal control subjects (an average of 2.3 ± 0.1 (standard error) pathogens) (**A**) and the distribution based on the quantitative cycle (Cq) cut-off values mentioned in Table 1 (**B**).

Stool specimens (370 each) from cases and controls were investigated using a multiplex qPCR panel assay to detect 24 target genes (Fig. 1A). The pathogen detection rates of bacteria, viruses, and parasites in diarrhea cases were 84.9%, 49.2%, and 3.2%, whereas the corresponding rates in the controls were 83.8%, 20.8%, and 4.9%, respectively. The numbers of *Escherichia coli* including enteroaggregative *E. coli* (EAEC) (*aggR, astA*) and enteropathogenic *E. coli* (EPEC) (*eae*) were relatively high in both cases and controls. *Shigella*/EIEC (odds ratio (OR) 2.40, *P* = 0.003), *Aeromonas* (OR 1.78, *P* = 0.008), *Campylobacter* (OR 4.21, *P* < 0.001), astrovirus (OR 5.57, *P* = 0.007), norovirus GII (OR 4.01, *P* < 0.001), and rotavirus (OR 31.94, *P* < 0.001) were detected more often in patients than in controls (Table 1). The detection rates of enteric adenovirus in the adenovirus-positive samples assessed using other qPCR assays^23^ were also not significant (OR 1.3, *P* = 0.393) (data not shown). EAEC (*astA*) (OR 0.64, *P* = 0.004) and *Plesiomonas* (OR 0.55, *P* = 0.013) were more common in non-diarrhea controls without an adjusted Cq cut-off value. An average of 2.7 ± 0.1 (standard error) pathogens was detected using qPCR in patients with diarrhea, whereas 2.3 ± 0.1 were detected in the controls (Fig. 2A). The percentages of “no pathogen detected” and mixed infection were 5.9% and 75.7% (IQR, 3; mean, 3.3) in cases, and 12.2% and 66.5% (IQR, 2; mean, 3.1) in controls, respectively.

**Table 1:** Detection of enteropathogen targets in stool samples from cases and control subjects, odds ratio, Cq cutoff value, and predicted causative agents by quantitative PCR analyses.

	Detection by quantitative PCR				Cq cutoff value	Detection based on optimal cut-off			
	Diarrhea (%)	Control (%)	OR	*P*-value		Diarrhea (%)	Control (%)	OR	*P*-value
Rotavirus	104 (28.1)	4 (1.1)	31.94	<0.001	27.98	80 (21.6)	0 (0)	205.34	<0.001
Norovirus GII	61 (16.5)	17 (4.6)	4.01	<0.001	20.23	29 (7.8)	0 (0)	64.01	<0.001
*Shigella/*EIEC	41 (11.1)	18 (4.9)	2.40	0.003	30.06	21 (5.7)	0 (0)	45.58	<0.001
*Campylobacter*	40 (10.8)	10 (2.7)	4.21	<0.001	28.97	18 (4.9)	0 (0)	38.89	<0.001
EAEC (*aggR*)	103 (27.8)	99 (26.8)	1.06	0.805	22.54	11 (3.0)	0 (0)	23.70	0.001
EAEC (*astA*)	221 (59.7)	259 (70.0)	0.64	0.004	19.95	10 (2.7)	1 (0.3)	7.17	0.011
*Aeromonas*	66 (17.8)	40 (10.8)	1.78	0.008	27.42	9 (2.4)	0 (0)	19.47	0.004
Sapovirus	15 (4.1)	10 (2.7)	1.50	0.416	27.20	9 (2.4)	0 (0)	19.47	0.004
Astrovirus	13 (3.5)	2 (0.5)	5.57	0.007	26.21	8 (2.2)	1 (0.3)	5.78	0.038
*Salmonella*	43 (11.6)	28 (7.6)	1.60	0.080	25.56	7 (1.9)	0 (0)	15.29	0.015
*Plesiomonas*	33 (8.9)	56 (15.1)	0.55	0.013	26.17	6 (1.6)	0 (0)	13.21	0.031
ETEC (*elt*)	35 (9.5)	40 (10.8)	0.86	0.626	25.29	6 (1.6)	0 (0)	13.21	0.031
	**Detection by quantitative PCR**				**Cq cutoff value**	**Detection based on optimal cut-off**			
	**Diarrhea (%)**	**Control (%)**	**OR**	***P*-value**		**Diarrhea (%)**	**Control (%)**	**OR**	***P*-value**
EPEC	125 (34.1)	149 (40.3)	0.77	0.094	21.08	5 (1.4)	0 (0)	11.15	0.062
ETEC (*st-p*)	19 (5.1)	11 (3.0)	1.73	0.191	26.24	4 (1.1)	0 (0)	9.10	0.124
Adenovirus	39 (10.5)	53 (14.3)	0.71	0.147	14.45	4 (1.1)	0 (0)	9.10	0.124
*Vibrio*	13 (3.5)	8 (2.2)	1.61	0.376	30.09	3 (0.8)	0 (0)	7.06	0.249
Norovirus GI	5 (1.4)	3 (0.8)	1.58	0.725	28.02	3 (0.8)	0 (0)	7.06	0.249
ETEC (*st-h*)	4 (1.1)	2 (0.5)	1.81	0.686	—	0 (0)	0 (0)	1	1
*Cryptosporidium*	8 (2.2)	8 (2.2)	1	1	—	0 (0)	0 (0)	1	1
*Giardia*	3 (0.8)	4 (1.1)	0.78	1	24.52	1 (0.3)	0 (0)	3.01	1
*Entamoeba*	1 (0.3)	6 (1.6)	0.23	0.123	—	0 (0)	0 (0)	1	1
*Yersinia*	2 (0.5)	7 (1.9)	0.33	0.177	—	0 (0)	0 (0)	1	1
STEC (*stx1*)	1 (0.3)	2 (0.5)	0.60	1	—	0 (0)	0 (0)	1	1
STEC (*stx2*)	1 (0.3)	0 (0)	3.01	1	37.74	1 (0.3)	0 (0)	3.01	1

The 12 targeted genes in 11 enteropathogens in the top half of the table were determined as the causative agents of acute diarrhea. Receiver operating curve (ROC) analyses were conducted to detect the optimal cut-off value. The cut-off value with the largest OR, which satisfied more than 95% specificity, was considered optimal. The relationships between case (control) and positive or negative control based on the calculated cut-off value were reported as ORs and P-values determined using the Fisher’s exact test.

Abbreviations: OR, odd ratio; Cq, quantification cycle; EIEC, Enteroinvasive *E. coli*; EAEC, Enteroaggregative *E. coli*; ETEC, Enterotoxigenic *E. coli*; EPEC, Enteropathogenic *E. coli*; STEC, Shiga toxin-producing *E. coli*.

### Cultivation and identification of bacterial enteropathogens isolated from stool specimens

Two hundred and thirty-four and 230 bacterial pathogens consisting of *Aeromonas caviae, A. hydrophila, A. sobria, A. veronii, Campylobacter coli, C. fetus, C. jejuni, E. coli*, *Plesiomonas shigelloides, Salmonella enterica*, *Shigella boydii, S. flexneri, S. sonnei, Vibrio fluvialis*, and *V. parahaemolyticus* were isolated from 170 cases (45.9%) and 169 controls (45.7%), respectively (Fig. 1B, Table 2). Multiple bacterial pathogens were isolated from 13.0% cases and 12.7% controls (Table 3). The most common bacteria were *E. coli* (31.4% vs 37.0%), *Salmonella* (8.9% vs 8.9%), and *Aeromonas* (5.9% vs 4.3%) in both cases and controls (Table 2). The isolation rates of *Shigella* and *Campylobacter* species were statistically associated with diarrhea, and *S. sonnei* and *C. jejuni* were the dominant species. Further, 306 isolates of *E. coli* were classified into 44 genotypes by multiplex-PCR assays and ≥8 different O-serotypes were identified among 66 *Salmonella* isolates. Most bacterial pathogens were similarly distributed in both patients and healthy control subjects.

**Table 2:** Bacterial pathogens isolated from cases and controls and their features.

Pathogen		Genotype or Serogroup	Number of isolates in cases	Number of isolates in controls
*Aeromonas*			23	18
	*A. caviae*		8	10
	*A. hydrophila*		4	0
	*A. sobria*		11	7
	*A. veronii*		0	1
*Campylobacter*			11	2
	*C. coli*		1	0
	*C. fetus*		1	0
	*C. jejuni*		9	2
*Escherichia*	*E. coli*		139	167
	EAEC		55	69
		*uidA, aafII, astA*	0	1
		*uidA, astA*, pCVD432	3	8
		*uidA, astA*, pCVD432*, pic*	3	0
		*uidA, astA, pic*	2	2
		*uidA, pic*	8	12
		*uidA*, pCVD432*, pic*	4	2
		*uidA*, pCVD432	10	20
		*astA*, pCVD432	0	2
		*pic*	2	2
		*uidA, aggR*	3	3
		*uidA, aggR, astA*	1	1
		*uidA, aggR, astA*, pCVD432	0	1
		*uidA, aggR, astA*, pCVD432*, pic*	2	5
		*uidA, aggR*, pCVD432	7	2
		*uidA, aggR*, pCVD432*, pic*	9	4
		*uidA, aggR, pic*	1	2
		*aggR, pic*, pCVD432	0	1
		*aggR*, pCVD432	0	1
	EPEC		39	42
		*uidA, eae*	32	24
		*uidA, eae, astA*	1	3
		*uidA, eae, bfpA*	1	1
		*uidA, eae, bfpB*	1	5
		*uidA, bfpB*	0	2
		*uidA, bfpB, astA*	0	1
		*eae*	4	6
	ETEC		14	9
		*uidA, elt*	3	1
		*uidA, elt, astA*	4	2
		*uidA, elt, est-p*	1	0
		*uidA, elt, est-p, astA*	0	1
		*uidA, est-h*	1	1
		*uidA, est-h, astA*	0	1
		*uidA, est-h, astA, bfpA*	0	1
		*uidA, est-p*	2	1
		*uidA, est-p, astA*	2	0
		*elt, est-p*	1	0
		*est-h, astA, bfpA*	0	1
	STEC	*uidA, stx1*	0	2*
	EIEC		3	3
		*uidA, ipaH, virF*	3	1
		*uidA, invE, astA*	0	1
		*invE*	0	1
	DAEC		1	5
		*uidA, daaE*	1	4
		*uidA, daaE, astA*	0	1
	Other		27	37
		*uidA, astA*	27	36
		*astA*	0	1
*Shigella*			11	1
	*S. boydii*		0	1
	*S. flexneri*		3	0
	*S. sonnei*		8	0
*Salmonella*	*S. enterica*		33	33
		O4	20	9
		O7	4	10
		O8	1	4
		O9	3	2
		O3,10	3	2
		O13	2	1
		O35	0	1
		Other O-antigen groups	0	4
*Plesiomonas*	*P. shigelloides*		13	9
*Vibrio*			4	0
	*V. fluvialis*		1	0
	*V. parahaemolyticus*		3	0

^*^Production of verotoxin-1 was confirmed by reverse passive latex agglutination, using the VTEC-RPLA kit (Denka Seiken, Japan).

**Table 3:** Number of bacterial pathogens isolated per subject.

Number of pathogen	Diarrhea	Control
0	200 (54.1%)	201 (54.3%)
1	122 (33.0%)	122 (33.0%)
2	33 (8.9%)	35 (9.5%)
3	14 (3.8%)	11 (3.0%)
4	1 (0.3%)	0 (0%)
5	0 (0%)	1 (0.3%)
Total	370 (100%)	370 (100%)

### Statistical analysis of qPCR data with the clinically relevant cut-off values

Due to the issues associated with high rates of asymptomatic carriage and the detection of multiple pathogens in patients, we next compared the quantification cycle values (Cq) of qPCR for each target gene in the case and control samples (Fig. S1). Cq cut-off values for each pathogen were determined using receiver operating curve (ROC) analysis (see Methods). Results showed that 12 targets had statistically significant associations with diarrhea occurrence (Table 1). With the clinically relevant cut-off values, pathogen-positive stool samples in control subjects were drastically reduced from 87.8% to 0.5%, whereas 181 diarrheal stools (48.9%) were positive for any of the 11 enteropathogens - rotavirus, norovirus GII, *Shigella*/enteroinvasive *E. coli* (EIEC), *Campylobacter*, EAEC, *Salmonella, Plesiomonas, Aeromonas*, sapovirus, astrovirus, and enterotoxigenic *E. coli* (ETEC) – that were strongly associated with acute diarrhea (Fig. 2B, Table 1). Moreover, rotavirus, norovirus GII, *Shigella*/enteroinvasive *E. coli* (EIEC), *Campylobacter*, EAEC, *Salmonella*, *Plesiomonas*, *Aeromonas*, sapovirus, astrovirus, and enterotoxigenic *E. coli* (ETEC) were strongly associated with acute diarrhea. Among 181 patients, single and multiple infections with the causative agents accounted for 84.0% and 16.0%, cases, respectively (Table 4). Mixed infection cases including bacterial–viral (7.7%, 14/181), bacterial–bacterial (4.4%, 8/181), and viral–viral (3.9%, 7/181) infections were still recognized despite the use of the stringent cut-off values. These mixed infection cases were observed more frequently in younger age groups (≤15 years of age) than in older age groups (≥16 years old) (*P* = 0.037).

**Table 4:** Number of cases and combinations of causative agents estimated among 181 patients.

Selected agents	Single infection (%)	Mixed infection		Total
		with one pathogen (cases)	with two pathogens (cases)	
Rotavirus	70	8	2	80
	(87.5)	astA* (3), Camp (2), NorII (2), Astro (1)	*aggR* & *astA* (1), Camp & Sal (1)	
Norovirus GII	20	8	1	29
	(69.0)	Camp (3), Astro (2), Rota (2), Sapo (1)	*aggR* & *astA* (1)	
*Shigella*/EIEC	17	4	0	21
	(81.0)	Sapo (1), Ples (1), *astA* (1), *elt* (1)		
*Campylobacter*	10	7	1	18
	(55.6)	NorII (3), Rota (2), Sapo (1), *aggR* (1)	Rota & Sal (1)	
EAEC (*aggR*)	6	3	2	11
	(54.5)	*astA* (1), Camp (1), *elt* (1)	*astA* & Rota (1), *astA* & NorII (1)	
EAEC (*astA*)	2	6	2	10
	(20.0)	Rota (3), *aggR* (1), Ples (1), Shig (1)	Rota & *aggR* (1), NorII & *aggR* (1)	
*Aeromonas*	8	1	0	9
	(88.9)	Ples (1)		
Sapovirus	4	5	0	9
	(44.4)	Shig (2), Camp (1), Astro (1), NorII (1)		
Astrovirus	4	4	0	8
	(50.0)	NorII (2), Rota (1), Sapo (1)		
*Salmonella*	5	0	2	7
	(71.4)		Rota & Camp (1), Ples & *elt* (1)	
*Plesiomonas*	3	2	1	6
	(50.0)	Aero (1), *astA* (1)	*elt* & Sal (1)	
ETEC (*elt*)	3	2	1	6
	(50.0)	*aggR* (1), Shig (1)	Ples & Sal (1)	

^*^astA; EAEC (*astA*), Camp; *Campylobacter*, Astro; astrovirus, *aggR*; EAEC (*aggR*), NorII; norovirus GII, Rota; rotavirus, Sapo; sapovirus, Ples; *Plesiomonas*, Shig; *Shigella*/EIEC, Aero; *Aeromonas*, Sal; *Salmonella, elt;* ETEC (*elt*).

### Patient features and clinical symptoms that might be caused by the detected pathogens

Patient features and clinical symptoms that might be caused by the detected pathogens were then investigated. Patients with a high frequency of defecation (more than 10 times per day) had significantly higher numbers of *Aeromonas* than all cases (*P* = 0.006) (Table S1). Rotavirus infection was significantly associated with fever (*P* = 0.034). Moreover, bacterial infections rather than viral infections tended to cause abdominal pain (*P* < 0.001). *Shigella*/EIEC (*P* = 0.020), *Salmonella* (*P* = 0.013), *Aeromonas* (*P* = 0.027), and *Plesiomonas* (*P* = 0.037) were more related to abdominal pain, whereas norovirus GII infection was rare in individuals with abdominal pain (*P* = 0.014). More than 20 WBCs/hpf (white blood cells per high power field) and red blood cells were present in the stool samples of patients with *Shigella*/EIEC infection (*P* < 0.001 and *P* = 0.012, respectively). Vomiting frequency was higher in rotavirus- (*P* < 0.001) and norovirus GII -infected patients (*P* = 0.009) than in all patients, but the incidence of nausea was significantly associated with *Aeromonas-* (*P* = 0.020) and sapovirus-infected patients (*P* = 0.037). Rotavirus (*P* = 0.001), norovirus GII (*P* = 0.004), and *Campylobacter* (*P* = 0.040) were more often detected in patients less than five years of age, whereas *Shigella/*EIEC (*P* = 0.001), *Aeromonas* (*P* = 0.010), and *Plesiomonas* (*P* = 0.021) were detected in patients older than five years of age (Table S2). The length of hospitalization for patients infected with each pathogen was not significantly different (*P* > 0.05). Pathogens that were associated with dry and cool seasons included rotavirus, followed by norovirus GII, whereas those associated with the other seasons (hot or rainy) included EAEC, *Shigella*/EIEC, and *Plesiomonas* (Table S3).

## Discussion

In this study, we investigated the etiological agents of acute diarrhea in patients with severe symptoms by quantitatively detecting a broad range of known enteropathogens. Our highly sensitive molecular assays, as well as bacteriological culture methods, detected high rates of asymptomatic cases and mixed infections in the study population. As the pathogen quantities in stool specimens from case and control subjects estimated using qPCR assays were different (Fig. S1), we set cut-off values of Cq for the specific diagnosis of patients and then predicted the causative pathogens of acute diarrhea. This considerably improved the assay and allowed differentiation between symptomatic and asymptomatic carriage; it also assisted in specifying disease-associated pathogens in the patients. Most illnesses were caused by rotavirus, followed by norovirus GII, *Shigella*/EIEC, and *Campylobacter*.

The Cq values obtained from qPCR assays for case and control samples were compared in each age group or each month (season) to identify trends and patterns of enteropathogen infections. The detection rates of *Shigella*/EIEC, *Aeromonas*, and *Plesiomonas* were higher in the ≥5 years of age group than in the <5 years of age group. In contrast, in addition to rotavirus, norovirus GII, and *Campylobacter*, ETEC, and sapovirus were likely to be more abundant in younger age groups (Table S2 and Fig. S3). These results might be due to differences in susceptibility to infections and/or lifestyles such as eating habits. Notably, EAEC (*aggR*) infection was also associated with diarrhea under the cut-off condition and nine of 11 patients were 1 year of age or under. Persistent diarrhea with EAEC is most frequently reported in children aged ≤1 year^24^. Our results thus indicate that infants are more susceptible to EAEC infection than older individuals. Furthermore, regarding asymptomatic infections with norovirus GII, 16 of 17 cases were less than 5 years of age and the remaining one was 6 years old. Infected asymptomatic carriers in young children might be important for the transmission of norovirus infection. Moreover, the seasonal trends of viral infections were clearer than those of bacterial infections (Table S3 and Fig. S2). Rotavirus, norovirus, and astrovirus were more likely to be abundant in the cool and dry season (November to February), whereas *Shigella*/EIEC, EAEC (*aggR*), and *Plesiomonas* were likely to be abundant in other seasons (rainy and/or hot). This approach can be useful for tracking the distribution and emergence of subclinically-infected persons, as well as diarrheal patients.

Next, we focused on the clinical symptoms of patients who were infected with the detected causative pathogens (Table S1). The patients infected with rotavirus presented with fever and mild to severe diarrhea. Vomiting was more common in rotavirus- and norovirus-infected patients than in all diarrhea patients; however, abdominal pain was less frequent in norovirus-infected patients (*P* = 0.014) than in rotavirus-infected patients (*P* = 0.139). In addition, rates of nausea in rotavirus- and norovirus-infected patients were 18/77 (23.4%) and 3/29 (10.3%), respectively. This might indicate a characteristic symptom of norovirus infection, that is, the sudden onset of vomiting. *Shigella/*EIEC, *Aeromonas*, *Salmonella*, and *Plesiomonas* infections were associated with abdominal pain. In patients positive for *Shigella*/EIEC, the number of fecal leucocytes (WBCs) and the appearance of erythrocytes (RBCs) in stool were significantly higher, which is one of the known characteristics of Shigellosis^25^. Clinical symptoms deduced from the qPCR results and patient data thus appear to correspond with the generally known symptoms caused by rotavirus, norovirus, or *Shigella*.

*Campylobacter* was also detected at significantly higher levels in diarrhea cases using qPCR and culture methods. This genus was likely to cause fever and unlikely to cause high frequent defecation in children. The role of *Aeromonas* as an etiological agent of acute diarrhea has been controversial^26,27^. Previously, in a challenge study, diarrhea was demonstrated in only two of 57 human volunteers, with doses ranging from 10^4^ to 10^10^ CFU^26^. In this study, many subclinical *Aeromonas* infections were recognized; however, such infections were associated with diarrhea in the cutoff condition and were accompanied by abdominal pain, nausea, and a high frequency of defecation. Thus, our analysis underscores the risk of illness caused by *Aeromonas*.

EAEC infection was not associated with any obvious characteristic clinical symptom. Investigations focusing on children (aged ≤1 year) who are probably highly susceptible to infection can be beneficial to understand this pathogen. Two genes, *astA* and *aggR*, were used in our qPCR panel assay to detect EAEC; however, *astA* was not always detected together with *aggR*. *astA* encodes EAST1 (EAEC heat-stable enterotoxin), which shares the functional properties of the enterotoxin (STa) secreted by ETEC^24^, whereas *aggR* is known as a transcriptional regulator (a key virulence regulator) and an important marker for virulent EAEC^28^. *astA* was detected not only in some isolates of EAEC, but also in isolates of EPEC, ETEC, EIEC, DAEC (Diffusely adherent *E. coli*), and non-categorized diarrheagenic *E. coli* (Table 2). Furthermore, single infection with *astA*-carrying agents was detected in only two of 10 cases (Table 4). These results imply that the presence of *astA* is not sufficient to conclusively identify the causative agents of diarrhea. Further, evaluation of the pathogenicity of these suspected pathogens will also be important.

This study has certain limitations. We could not determine the causative agents in approximately 50% of cases, although many enteropathogens were detected. Our estimates of causative agents were based on differences in pathogen loads between patient and control stool samples. In reality, the association between pathogen quantity and disease is host-pathogen-specific. In addition, pathogen quantities change in each stage of clinical manifestation^8,29^. These issues should be addressed in future studies to accurately predict etiological agents of disease. Further, the lower detection rates of certain enteropathogens such as *Vibrio*, norovirus GI, and STEC were not sufficient to interpret the qPCR results in this study. Further, the lack of consideration of matching pairs in the statistical analysis is also a limitation.

In a recent report, travelers’ diarrhea in foreign visitors to Thailand was investigated using a TaqMan array card assay^30^. One hundred and seventy-three cases from the in-patient or out-patient department in a private hospital and 165 non-diarrheal subjects were enrolled in that study. The results of that study were in agreement with our results regarding the high detection rates of *Campylobacter*, EAEC, EPEC, and norovirus GII, and the extremely low detection rates of protozoa including *Cryptosporidium*, *Entamoeba histolytica*, and *Giardia*. In contrast, rotavirus, adenovirus, and astrovirus were rarely detected in their study. The low detection rate of rotavirus might be due to the age of their enrolled patients, who were more than 18 years of age. Accordingly, massive surveillance using several approaches will assist in identifying a panel of microbial pathogens associated with acute diarrhea.

In conclusion, the causative agents of acute diarrhea were predicted using a multiplex qPCR panel assay with patient-specific cut-off values, and the characteristic clinical symptoms caused by each enteropathogen were partially revealed. This approach can provide new insights into infectious diarrheal diseases. Data accumulation, validation of the results, and the re-design/adjustment for known or unknown target enteropathogens in a multiplex quantitative assay could be critical to establish superior diagnostic procedures.

## Methods

### Specimen collection

Stool specimens were collected from patients hospitalized with acute diarrhea from eight Thai government hospitals nationwide during April 2016 to March 2018 (Table S4). Of all cases, 353 (95.4%) were patients with abnormally loose stools more than three times within 24 h and a diarrhea-free time of at least 7 days prior to the collection of stool specimens. The remaining 4.6% cases were associated with less-frequent defecation. Eligible cases were excluded from the study if they had known immunodeficiency or chronic causes of diarrheal symptoms, or if stool volume was too small (<3 g) for the experiments. Stool specimens were collected from patients prior to starting the antibiotic treatment in the hospitals or immediately after antibiotic treatment (<1 h). For each diarrhea case, one healthy volunteer who had no history of diarrhea for at least 30 days before enrolment and who was a match with the case regarding age (mostly within 3 years of the age of the case) and area of residence (living in at least the same province) was selected among the residents or visitors to the hospital by public health officers, coordinators, or nurses. Stool specimens collected in a sterile container were first stored at 4–10 °C at each site and shipped overnight on ice packs to the central reference laboratory of the National Institute of Health (NIH) of Thailand.

### Culture methods

Stool samples collected from diarrheal and non-diarrheal control subjects were cultured as part of routine work—for example, direct plating/selective enrichment, isolation, and identification by biochemical tests according to the procedure of the NIH of Thailand. The viable enteric bacteria including *Salmonella* spp., *Shigella* spp., *Vibrio* spp., *Aeromonas* spp., *P. shigelloides*, *E. coli*, and *Campylobacter* spp. were isolated. Some isolates of enteropathogens were further confirmed or characterized by PCR and serotyping. Five colonies of *E. coli* on MacConkey agar, sorbitol MacConkey agar, Salmonella-Shigella agar, or xylose lysine deoxycholate agar were examined by multiplex-PCR assays^31–34^ for the detection and differentiation of pathogenic *E. coli*. For the isolation of *Campylobacter* spp., 1–2 g of stool was enriched in 2 ml Preston broth at 37 °C for 3–4 h before dropping stool suspensions on modified charcoal, cefoperazone, deoxycholate agar (mCCDA) and incubating them at 37 °C for 48 h in anaerobic jars under microaerophilic conditions (Anaero Pack-MicroAero, Mitsubishi Gas Chemical, Japan).

### Quantitative PCR assay

We used TaqMan real-time PCR-based assays for the simultaneous detection of enteropathogens, namely astrovirus (ORF1a), sapovirus (polymerase/capsid junction), adenovirus (hexon), norovirus GI (RdRp/capsid junction), norovirus GII (ORF1-ORF2), rotavirus group A (NSP3), *Cryptosporidium* spp. (COWP), *Giardia lamblia* (ITS1), *E. histolytica* (18S rRNA), *P. shigelloides* (*hugA*), *Campylobacter* spp. (*gyrB*), *Vibrio* spp. (*toxR*), *Salmonella* spp. (*invA*), *Aeromonas* spp. (*aerA*), *Yersinia* spp. (*lysP*), *Shigella*/EIEC (*ipaH*), EPEC (*eae*), ETEC (*elt*, *est-h*, *est-p*), EAEC (*aggR*, *astA*), and Shiga toxin-producing *E. coli* (STEC) (*stx*1, *stx*2), following the detection procedure described previously^22^. Several of the defining markers of *E. coli* pathotypes are proven virulence determinants of the respective pathotype, but for EAEC, the essential virulence determinant has not been proven^35^.

Total RNA and DNA were extracted from individual stool samples using QIAamp viral RNA and QIAamp fast DNA stool mini kits (cat#52906 and #51604, Qiagen, USA), respectively, either manually or on a robotic workstation for the automated purification of nucleic acids. For viral RNA/DNA preparation, whole stool (200 mg) was suspended in 1.8 ml saline, which was centrifuged at 4,000 × *g* for 20 min and the supernatant containing viral particles was separated. For bacterial and parasitic DNA preparation, diarrheal stool samples were centrifuged at 15,000 × *g* for 1 min to obtain a stool pellet (wet weight, 200 mg). qPCR assays were performed using an Applied Biosystems 7500 Fast real*-*time PCR system (Foster City, CA, USA). Target genes were amplified using the QuantiFast Pathogen RT-PCR and PCR kits, respectively. Herein, pure water was used as an extraction control to monitor DNA cross-contamination and for environmental contaminants during extraction. For preventing laboratory contamination, preparation of the PCR master mix, extraction of DNA/RNA from stool specimens, addition of DNA/RNA templates to the master mix, and qPCR reactions were conducted in separate areas/rooms in our laboratory. Moreover, each batch of the master mix for real-time PCR reaction was always tested to monitor contamination, along with the test samples.

qPCR data were interpreted according to the following criteria: (i) quantification cycle (Cq) value, defined as the number of PCR cycles where the fluorescent signal exceeded the detection threshold, which was fixed at 0.2, 0.3, and 0.5 relative fluorescence units for viral, parasitic, and bacterial targets, respectively; (ii) greater than or equal to a Cq value of 41 was considered as negative for all pathogens in this study; (iii) signals from the internal control (IC) RNA and IC DNA had Cq values of 31 ± 3 and 32 ± 3, respectively; (iv) positive and no template controls (NTC) were used and verified for validity in every qPCR run^22^.

### Statistical analysis

We reported the number of detections and their percentage for each pathogen by case and control groups. We also estimated the ORs and performed Fisher’s exact tests. The complex infections were counted and reported as proportions. For each pathogen, Cq values of qPCR-positive samples were presented as median, interquartile range (IQR), and total range. ROC analyses were conducted to detect the optimal cut-off value. The cut-off value with the largest OR, which satisfied more than 95% specificity, was considered optimal. In all procedures, we calculated the OR based on the contingency table by adding a 0.5 correction value to all cells (Haldane-Anscombe 1/2 correction) to address zero- cell in the two-by-two contingency table for case/control and positive/negative. The relationships between case (control) and positive or negative controls based on the calculated cut-off value were reported as ORs and *P*-values, as determined using the Fisher’s exact test. All statistical analyses were performed using R (The R Foundation for Statistical Computing, ver. 3.5.1) and all tests were two-tailed. *P*-values < 0.05 were considered statistically significant.

### Ethical statements

The clinical protocol was approved by the Ethical Review Committee for Research in Human Subjects, Ministry of Public Health, Thailand (reference no. 44/2558), Institutional Review Board BIDI (S023q/58), Prapokklao Hospital (CTIREC044), Sunprasitthiprasong Hospital (063/2559), Chiang Rai Prachanukroh Hospital (CR 0032.102/9844), and Institutional Review Board of Maesot General Hospital and Samutsakhon Hospital. Stool specimens were collected from all the volunteers who provided written informed consent. The methods were carried out in accordance with the approved guidelines.

## Supplementary information

Table S1-S4, Figure S1-S3, Methods.
