# Supplementary material for: Etiologic features of diarrheagenic microbes in stool specimens from patients with acute diarrhea in Thailand
Source: Sci Rep. 2020 Mar 4;10:4009. doi: 10.1038/s41598-020-60711-1 (PMC7055299; doi:10.1038/s41598-020-60711-1)
Supplement: Supplementary file 1 — Table S1-S4, Figure S1-S3, Methods. [file 41598_2020_60711_MOESM1_ESM.pdf]

Etiologic features of diarrheagenic microbes in stool specimens from patients with acute diarrhea in Thailand

Kazuhisa Okada[1,2]*, Warawan Wongboot[1,3], Watcharaporn Kamjumphol[1], Namfon Suebwongsa[1], Piyada Wangroongsarb[3], Pipat Kluabwang[4], Nuttagarn Chuenchom[5], Witaya Swaddiwudhipong[6], Thanee Wongchai[7], Weerawat Manosuthi[8], Norrathep Assawapatchara[9], Patchanee Khum-on[10], Patpong Udompat[11], Chareeya Thanee[12], Suwatthiya Kitsaran[13], Lakkana Jirapong[14], Charoen Jaiwong[15], Supalert Nedsuwan[16], Chotipong Siripipattanamongkol[17], Pilailuk Akkapaiboon Okada[3], Siriporn Chantaroj[3], Sho Komukai[18], Shigeyuki Hamada[1,2]

[1]Thailand-Japan Research Collaboration Center on Emerging and Re-emerging Infections, Nonthaburi, Thailand; [2]Research Institute for Microbial Diseases, Osaka University, Osaka, Japan; [3]National Institute of Health, Department of Medical Sciences, Nonthaburi, Thailand; [4]Department of Pediatrics, [5]Department of Medicine, [6]Department of Community and Social Medicine, and [7]Department of Clinical Laboratory, Maesot General Hospital, Tak, Thailand; [8]Department of Medicine, Bamrasnaradura Infectious Diseases Institute, Nonthaburi, Thailand; [9]Department of Medicine, Ranong Hospital, Ranong, Thailand; [10]Department of Medical Technology, Chum Phae Hospital, Khon Kaen, Thailand; [11]Department of Community and Social Medicine, Prapokklao Hospital, Chanthaburi, Thailand; [12]Department of Pediatrics and [13]Department of Medicine, Sunpasitthiprasong Hospital, Ubon Ratchathani, Thailand; [14]Department of Radiology, Samutsakhon Hospital, Samutsakhon, Thailand; [15]Department of Pediatrics, [16]Department of Preventive and Social Medicine, and [17]Department of Medicine, Chiangrai Prachanukroh Hospital, Chiang Rai, Thailand; and [18]Department of Integrated Medicine of Graduate school of Medicine, Osaka University, Osaka, Japan

***Corresponding author:** Kazuhisa Okada
E-mail Address: kazuhisa@biken.osaka-u.ac.jp

Supplemental material

Table S1
Table S2
Table S3
Table S4
Figure S1
Figure S2
Figure S3
Methods

Figure S1. Box and whisker plot showing quantitation cycle (Cq) values of qPCR-positive samples for 24 target genes among 370 diarrheal and 370 non-diarrheal control subjects. The bottom and top of each box represent 25th and 75th percentiles of the data, respectively. The median value is displayed inside the box by the line. The maximum and minimum values are displayed with vertical lines connecting the points to the center box, along with the outliers represented herein indicated by closed and open circles. Numbers indicate the number of positive specimens.

Figure S2. Detection of enteropathogens by month based on qPCR assays and distribution of Cq-values. The blue dots represent Cq values from the non-diarrheal control subjects and the red dots represent Cq values from the diarrheal patients. The red line indicates the cut-off value shown in Table 1.

Figure S3. Detection of enteropathogens by age group using qPCR assays and distribution of Cq-values. The blue dots represent Cq values from the non-diarrheal control subjects and the red dots represent Cq values from the diarrheal patients. The red line indicates the cut-off value shown in Table 1.

Table S1. Clinical symptoms caused by each enteropathogen as determined based on quantitative PCR results and patient data

	All cases					Cases under Cq-cutoff value				
**Rotavirus**	**Yes**	**No**	**OR**	***P*-value**		**Yes**	**No**	**OR**	***P*-value**	
Fever	90 (32.6)	11 (12.9)	3.14 (1.61-6.14)	< 0.001	***	66 (23.9)	11 (12.9)	2.05 (1.04-4.04)	0.034	*
Nausea	19 (22.4)	82 (29.7)	0.69 (0.39-1.22)	0.215		18 (21.2)	59 (21.4)	1 (0.56-1.81)	1	
Vomiting	74 (34.3)	27 (18.6)	2.25 (1.37-3.72)	0.001	**	62 (28.7)	15 (10.3)	3.41 (1.86-6.22)	< 0.001	***
Diarrhea (≥ 10 times/day)	30 (31.6)	69 (27.1)	1.25 (0.75-2.08)	0.425		21 (22.1)	54 (21.2)	1.07 (0.61-1.88)	0.884	
Abdominal pain	18 (19.8)	83 (30.7)	0.57 (0.32-1)	0.058		14 (15.4)	63 (23.3)	0.61 (0.33-1.14)	0.139	
Fecal WBC	3 (18.8)	25 (23.8)	0.82 (0.23-2.88)	0.762		1 (6.2)	18 (17.1)	0.46 (0.08-2.63)	0.462	
Fecal RBC	6 (35.3)	19 (26.8)	1.52 (0.51-4.54)	0.553		4 (23.5)	14 (19.7)	1.32 (0.39-4.44)	0.743	
Hospital stay			-0.66 (-1.45, 0.13)	0.105				-0.57 (-1.47, 0.32)	0.210	
**Norovirus GII**	**Yes**	**No**	**OR**	***P*-value**		**Yes**	**No**	**OR**	***P*-value**	
Fever	44 (15.9)	15 (17.6)	0.87 (0.46-1.65)	0.738		20 (7.2)	9 (10.6)	0.64 (0.29-1.45)	0.361	
Nausea	11 (12.9)	48 (17.4)	0.73 (0.36-1.46)	0.403		3 (3.5)	26 (9.4)	0.4 (0.13-1.26)	0.108	
Vomiting	47 (21.8)	12 (8.3)	2.99 (1.54-5.81)	0.001	**	24 (11.1)	5 (3.4)	3.25 (1.26-8.41)	0.009	**
Diarrhea (≥ 10 times/day)	11 (11.6)	45 (17.6)	0.63 (0.31-1.26)	0.192		5 (5.3)	22 (8.6)	0.63 (0.24-1.65)	0.371	
Abdominal pain	7 (7.7)	52 (19.3)	0.37 (0.17-0.83)	0.009	**	2 (2.2)	27 (10.0)	0.25 (0.07-0.92)	0.014	*
Fecal WBC	3 (18.8)	21 (20.0)	1.02 (0.29-3.62)	1		1 (6.2)	10 (9.5)	0.88 (0.15-5.29)	1	
Fecal RBC	1 (5.9)	20 (28.2)	0.23 (0.04-1.31)	0.062		1 (5.9)	10 (14.1)	0.53 (0.09-3.21)	0.684	
Hospital stay			-0.61 (-1.58, 0.37)	0.223				-0.28 (-1.62, 1.06)	0.683	
***Shigella*/EIEC**	**Yes**	**No**	**OR**	***P*-value**		**Yes**	**No**	**OR**	***P*-value**	
Fever	32 (11.6)	9 (10.6)	1.07 (0.5-2.31)	1		16 (5.8)	5 (5.9)	0.93 (0.34-2.51)	1	
Nausea	10 (11.8)	31 (11.2)	1.08 (0.51-2.28)	0.847		8 (9.4)	13 (4.7)	2.14 (0.87-5.24)	0.115	
Vomiting	25 (11.6)	16 (11.0)	1.05 (0.54-2.02)	1		13 (6.0)	8 (5.5)	1.07 (0.44-2.6)	1	
Diarrhea (≥ 10 times/day)	8 (8.4)	32 (12.5)	0.67 (0.3-1.48)	0.347		5 (5.3)	15 (5.9)	0.94 (0.35-2.57)	1	
Abdominal pain	14 (15.4)	27 (10.0)	1.66 (0.83-3.29)	0.182		10 (11.0)	11 (4.1)	2.91 (1.21-6.96)	0.020	*
Fecal WBC	7 (43.8)	9 (8.6)	8.02 (2.5-25.75)	0.001	**	7 (43.8)	2 (1.9)	32.68 (6.75-158.38)	< 0.001	***
Fecal RBC	5 (29.4)	9 (12.7)	2.89 (0.86-9.74)	0.134		5 (29.4)	4 (5.6)	6.6 (1.65-26.35)	0.012	*
Hospital stay			-0.31 (-1.41, 0.79)	0.580				-0.74 (-2.23, 0.74)	0.329	
Campylobacter	**Yes**	**No**	**OR**	***P*-value**		**Yes**	**No**	**OR**	***P*-value**	
Fever	36 (13.0)	4 (4.7)	2.75 (1-7.55)	0.031	*	16 (5.8)	2 (2.4)	2.12 (0.55-8.18)	0.263	
Nausea	9 (10.6)	31 (11.2)	0.97 (0.45-2.09)	1		4 (4.7)	14 (5.1)	1 (0.34-2.96)	1	
Vomiting	20 (9.3)	20 (13.8)	0.64 (0.33-1.23)	0.231		9 (4.2)	9 (6.2)	0.66 (0.26-1.66)	0.461	
Diarrhea (≥ 10 times/day)	3 (3.2)	36 (14.1)	0.23 (0.07-0.7)	0.003	**	1 (1.1)	17 (6.7)	0.22 (0.04-1.17)	0.032	*
Abdominal pain	7 (7.7)	33 (12.2)	0.63 (0.27-1.44)	0.334		2 (2.2)	16 (5.9)	0.43 (0.11-1.67)	0.263	
Fecal WBC	1 (6.2)	14 (13.3)	0.61 (0.1-3.57)	0.690		1 (6.2)	9 (8.6)	0.98 (0.16-5.97)	1	
Fecal RBC	3 (17.6)	6 (8.5)	2.43 (0.59-10.05)	0.367		3 (17.6)	5 (7.0)	2.92 (0.68-12.51)	0.180	
Hospital stay			0.96 (-0.13, 2.06)	0.086				0.72 (-0.76, 2.21)	0.342	
**EAEC (*aggR*)**	**Yes**	**No**	**OR**	***P*-value**		**Yes**	**No**	**OR**	***P*-value**	
Fever	74 (26.8)	27 (31.8)	0.78 (0.46-1.32)	0.408		9 (3.3)	2 (2.4)	1.19 (0.29-4.88)	1	
Nausea	22 (25.9)	79 (28.6)	0.88 (0.51-1.52)	0.680		3 (3.5)	8 (2.9)	1.34 (0.38-4.77)	0.725	
Vomiting	52 (24.1)	49 (33.8)	0.62 (0.39-0.99)	0.055		5 (2.3)	6 (4.1)	0.56 (0.18-1.77)	0.360	
Diarrhea (≥ 10 times/day)	23 (24.2)	70 (27.5)	0.85 (0.5-1.46)	0.588		1 (1.1)	10 (3.9)	0.37 (0.07-2.09)	0.301	
Abdominal pain	27 (29.7)	74 (27.4)	1.12 (0.67-1.89)	0.687		1 (1.1)	10 (3.7)	0.41 (0.07-2.31)	0.303	
Fecal WBC	5 (31.2)	25 (23.8)	1.51 (0.5-4.58)	0.541		1 (6.2)	4 (3.8)	2.18 (0.32-14.94)	0.514	
Fecal RBC	6 (35.3)	17 (23.9)	1.76 (0.58-5.3)	0.366		1 (5.9)	4 (5.6)	1.36 (0.2-9.35)	1	
Hospital stay			-0.41 (-1.19, 0.38)	0.311				-1.07 (-3.28, 1.13)	0.341	
**EAEC (*astA*)**	**Yes**	**No**	**OR**	***P*-value**		**Yes**	**No**	**OR**	***P*-value**	
Fever	163 (59.1)	54 (63.5)	0.83 (0.51-1.37)	0.527		7 (2.5)	3 (3.5)	0.66 (0.18-2.39)	0.705	
Nausea	52 (61.2)	165 (59.8)	1.06 (0.64-1.73)	0.899		2 (2.4)	8 (2.9)	0.95 (0.23-3.96)	1	
Vomiting	129 (59.7)	88 (60.7)	0.96 (0.63-1.48)	0.913		7 (3.2)	3 (2.1)	1.46 (0.4-5.28)	0.746	
Diarrhea (≥ 10 times/day)	56 (58.9)	152 (59.6)	0.97 (0.6-1.56)	1		1 (1.1)	8 (3.1)	0.46 (0.08-2.66)	0.454	
Abdominal pain	61 (67.0)	156 (57.8)	1.48 (0.9-2.42)	0.138		1 (1.1)	9 (3.3)	0.46 (0.08-2.6)	0.462	
Fecal WBC	10 (62.5)	59 (56.2)	1.26 (0.44-3.61)	0.788		0 (0.0)	4 (3.8)	0.68 (0.04-13.29)	1	

Fecal RBC	10 (58.8)	38 (53.5)	1.22 (0.43-3.46)	0.790		1 (5.9)	3 (4.2)	1.78 (0.24-12.98)	1	
Hospital stay			-0.65 (-1.36, 0.06)	0.072				0.65 (-1.95, 3.26)	0.624	
Aeromonas	**Yes**	**No**	**OR**	***P*-value**		**Yes**	**No**	**OR**	***P*-value**	
Fever	44 (15.9)	19 (22.4)	0.65 (0.36-1.19)	0.192		4 (1.4)	4 (4.7)	0.3 (0.08-1.13)	0.092	
Nausea	25 (29.4)	38 (13.8)	2.61 (1.47-4.64)	0.002	**	5 (5.9)	3 (1.1)	5.34 (1.37-20.86)	0.020	*
Vomiting	38 (17.6)	25 (17.2)	1.02 (0.59-1.77)	1		6 (2.8)	2 (1.4)	1.77 (0.41-7.74)	0.483	
Diarrhea (≥ 10 times/day)	27 (28.4)	34 (13.3)	2.58 (1.46-4.56)	0.001	**	6 (6.3)	2 (0.8)	7.36 (1.68-32.3)	0.006	**
Abdominal pain	25 (27.5)	38 (14.1)	2.32 (1.31-4.09)	0.006	**	5 (5.5)	3 (1.1)	4.86 (1.24-18.97)	0.027	*
Fecal WBC	3 (18.8)	27 (25.7)	0.74 (0.21-2.59)	0.758		0 (0.0)	5 (4.8)	0.55 (0.03-10.49)	1	
Fecal RBC	4 (23.5)	17 (23.9)	1.04 (0.31-3.43)	1		1 (5.9)	3 (4.2)	1.78 (0.24-12.98)	1	
Hospital stay			0.16 (-0.76, 1.09)	0.731				-0.16 (-2.77, 2.44)	0.903	
**Sapovirus**	**Yes**	**No**	**OR**	***P*-value**		**Yes**	**No**	**OR**	***P*-value**	
Fever	11 (4.0)	4 (4.7)	0.78 (0.26-2.4)	0.759		7 (2.5)	2 (2.4)	0.93 (0.22-3.97)	1	
Nausea	7 (8.2)	8 (2.9)	3.02 (1.1-8.32)	0.055		5 (5.9)	4 (1.4)	4.14 (1.16-14.74)	0.037	*
Vomiting	11 (5.1)	4 (2.8)	1.76 (0.58-5.34)	0.421		6 (2.8)	3 (2.1)	1.26 (0.34-4.69)	0.745	
Diarrhea (≥ 10 times/day)	1 (1.1)	13 (5.1)	0.29 (0.05-1.57)	0.124		1 (1.1)	7 (2.7)	0.53 (0.09-3.08)	0.688	
Abdominal pain	2 (2.2)	13 (4.8)	0.53 (0.14-2.1)	0.373		2 (2.2)	7 (2.6)	0.98 (0.23-4.19)	1	
Fecal WBC	0 (0.0)	3 (2.9)	0.89 (0.04-17.98)	1		0 (0.0)	2 (1.9)	1.25 (0.06-27.31)	1	
Fecal RBC	1 (5.9)	1 (1.4)	4.27 (0.42-43.73)	0.351		1 (5.9)	0 (0.0)	13 (0.51-333.64)	0.193	
Hospital stay			-0.33 (-2.28, 1.63)	0.744				-0.37 (-2.97, 2.24)	0.783	
**Astrovirus**	**Yes**	**No**	**OR**	***P*-value**		**Yes**	**No**	**OR**	***P*-value**	
Fever	9 (3.3)	2 (2.4)	1.19 (0.29-4.88)	1		6 (2.2)	2 (2.4)	0.8 (0.18-3.52)	1	
Nausea	2 (2.4)	9 (3.3)	0.84 (0.2-3.47)	1		2 (2.4)	6 (2.2)	1.25 (0.28-5.47)	1	
Vomiting	7 (3.2)	4 (2.8)	1.13 (0.34-3.69)	1		5 (2.3)	3 (2.1)	1.06 (0.27-4.11)	1	
Diarrhea (≥ 10 times/day)	4 (4.2)	7 (2.7)	1.63 (0.49-5.37)	0.498		3 (3.2)	5 (2.0)	1.72 (0.44-6.72)	0.452	
Abdominal pain	2 (2.2)	9 (3.3)	0.77 (0.19-3.16)	0.737		2 (2.2)	6 (2.2)	1.14 (0.26-4.98)	1	
Fecal WBC	1 (6.2)	3 (2.9)	2.83 (0.39-20.65)	0.437		1 (6.2)	1 (1.0)	6.74 (0.66-68.96)	0.248	
Fecal RBC	0 (0.0)	1 (1.4)	1.34 (0.05-34.4)	1		0 (0.0)	1 (1.4)	1.34 (0.05-34.4)	1	
Hospital stay			-0.93 (-3.13, 1.28)	0.412				-1.02 (-3.93, 1.88)	0.49	
Salmonella	**Yes**	**No**	**OR**	***P*-value**		**Yes**	**No**	**OR**	***P*-value**	
Fever	34 (12.3)	9 (10.6)	1.15 (0.53-2.46)	0.848		7 (2.5)	0 (0.0)	4.76 (0.27-84.19)	0.206	
Nausea	8 (9.4)	35 (12.7)	0.75 (0.34-1.65)	0.565		2 (2.4)	5 (1.8)	1.48 (0.32-6.72)	0.670	
Vomiting	22 (10.2)	21 (14.5)	0.67 (0.36-1.26)	0.247		4 (1.9)	3 (2.1)	0.86 (0.21-3.54)	1	
Diarrhea (≥ 10 times/day)	20 (21.1)	22 (8.6)	2.82 (1.47-5.41)	0.003	**	3 (3.2)	4 (1.6)	2.11 (0.51-8.72)	0.395	
Abdominal pain	12 (13.2)	31 (11.5)	1.2 (0.59-2.41)	0.709		5 (5.5)	2 (0.7)	6.83 (1.5-31.05)	0.013	*
Fecal WBC	3 (18.8)	11 (10.5)	2.13 (0.57-8.02)	0.396		0 (0.0)	3 (2.9)	0.89 (0.04-17.98)	1	
Fecal RBC	4 (23.5)	8 (11.3)	2.49 (0.69-9)	0.236		0 (0.0)	1 (1.4)	1.34 (0.05-34.4)	1	
Hospital stay			-0.59 (-1.66, 0.48)	0.282				-0.01 (-2.92, 2.9)	0.995	
Plesiomonas	**Yes**	**No**	**OR**	***P*-value**		**Yes**	**No**	**OR**	***P*-value**	
Fever	18 (6.5)	14 (16.5)	0.35 (0.17-0.74)	0.008	**	3 (1.1)	3 (3.5)	0.3 (0.07-1.35)	0.145	
Nausea	10 (11.8)	22 (8.0)	1.57 (0.72-3.42)	0.281		1 (1.2)	5 (1.8)	0.88 (0.14-5.42)	1	
Vomiting	21 (9.7)	11 (7.6)	1.29 (0.61-2.72)	0.573		2 (0.9)	4 (2.8)	0.37 (0.08-1.75)	0.224	
Diarrhea (≥ 10 times/day)	9 (9.5)	22 (8.6)	1.14 (0.51-2.53)	0.833		0 (0.0)	6 (2.4)	0.2 (0.01-3.6)	0.196	
Abdominal pain	18 (19.8)	14 (5.2)	4.45 (2.14-9.28)	< 0.001	***	4 (4.4)	2 (0.7)	5.52 (1.16-26.41)	0.037	*
Fecal WBC	1 (6.2)	15 (14.3)	0.57 (0.1-3.29)	0.692		0 (0.0)	4 (3.8)	0.68 (0.04-13.29)	1	
Fecal RBC	1 (5.9)	9 (12.7)	0.6 (0.1-3.64)	0.679		0 (0.0)	2 (2.8)	0.79 (0.04-17.31)	1	
Hospital stay			-0.19 (-1.43, 1.04)	0.759				-0.98 (-3.58, 1.63)	0.462	
**ETEC (*elt*)**	**Yes**	**No**	**OR**	***P*-value**		**Yes**	**No**	**OR**	***P*-value**	
Fever	27 (9.8)	7 (8.2)	1.15 (0.49-2.69)	0.832		4 (1.4)	2 (2.4)	0.55 (0.12-2.64)	0.629	
Nausea	8 (9.4)	26 (9.4)	1.04 (0.46-2.34)	1		3 (3.5)	3 (1.1)	3.32 (0.74-14.89)	0.145	
Vomiting	21 (9.7)	13 (9.0)	1.08 (0.53-2.21)	0.856		2 (0.9)	4 (2.8)	0.37 (0.08-1.75)	0.224	
Diarrhea (≥ 10 times/day)	10 (10.5)	24 (9.4)	1.16 (0.54-2.49)	0.839		3 (3.2)	3 (1.2)	2.73 (0.61-12.24)	0.350	
Abdominal pain	7 (7.7)	27 (10.0)	0.79 (0.34-1.83)	0.678		2 (2.2)	4 (1.5)	1.65 (0.35-7.91)	0.644	
Fecal WBC	3 (18.8)	8 (7.6)	2.97 (0.76-11.69)	0.161		0 (0.0)	1 (1.0)	2.11 (0.08-54.04)	1	
Fecal RBC	3 (17.6)	5 (7.0)	2.92 (0.68-12.51)	0.180		0 (0.0)	0 (0.0)	4.09 (0.08-213.19)	1	
Hospital stay			-0.37 (-1.56, 0.82)	0.540				-1.28 (-4.18, 1.62)	0.389	

*, $P < 0.05$; **, $P < 0.01$; ***, $P < 0.001$.

Table S2. Detection of enteropathogens in patients under and over five years of age using qPCR assays

	Number of positive (%) in all cases					Number of positive (%) in cases under Cq-cutoff value				
	< 5 yrs	≥ 5 yrs	OR	*P* -value		< 5 yrs	≥ 5 yrs	OR	*P* -value	
**Rotavirus**	83 (34.4)	21 (16.3)	0.38 (0.22-0.64)	< 0.001	***	64 (26.6)	16 (12.4)	0.4 (0.22-0.72)	0.001	**
**Norovirus GII**	50 (20.7)	11 (8.5)	0.37 (0.19-0.73)	0.003	**	26 (10.8)	3 (2.3)	0.22 (0.07-0.7)	0.004	**
***Shigella* /EIEC**	21 (8.7)	20 (15.5)	1.92 (1.01-3.67)	0.056		6 (2.5)	15 (11.6)	4.9 (1.91-12.59)	0.001	**
Campylobacter	30 (12.4)	10 (7.8)	0.61 (0.29-1.27)	0.218		16 (6.6)	2 (1.6)	0.27 (0.07-1.03)	0.040	*
**EAEC (*aggR*)**	64 (26.6)	39 (30.2)	1.2 (0.75-1.92)	0.467		10 (4.1)	1 (0.8)	0.26 (0.05-1.44)	0.106	
**EAEC (*astA*)**	133 (55.2)	88 (68.2)	1.73 (1.11-2.71)	0.019	*	8 (3.3)	2 (1.6)	0.54 (0.13-2.24)	0.504	
Aeromonas	34 (14.1)	32 (24.8)	2 (1.17-3.43)	0.015	*	2 (0.8)	7 (5.4)	5.87 (1.38-24.95)	0.010	*
**Sapovirus**	13 (5.4)	2 (1.6)	0.33 (0.08-1.3)	0.097		7 (2.9)	2 (1.6)	0.61 (0.14-2.61)	0.505	
**Astrovirus**	8 (3.3)	5 (3.9)	1.21 (0.41-3.63)	0.774		5 (2.1)	3 (2.3)	1.19 (0.31-4.62)	1	
Salmonella	31 (12.9)	12 (9.3)	0.71 (0.36-1.42)	0.395		3 (1.2)	4 (3.1)	2.44 (0.59-10.04)	0.244	
Plesiomonas	11 (4.6)	22 (17.1)	4.2 (1.99-8.85)	< 0.001	***	1 (0.4)	5 (3.9)	7.08 (1.15-43.64)	0.021	*
**ETEC (*elt*)**	29 (12.0)	6 (4.7)	0.38 (0.16-0.91)	0.024	*	4 (1.7)	2 (1.6)	1.03 (0.22-4.93)	1	

*, $P < 0.05$; **, $P < 0.01$; ***, $P < 0.001$.

Table S3. Number of pathogens detected in each month or season based on qPCR in 370 diarrheal cases

	**Hot**				**Rainy**				**Dry and cool**				***P*-value**	
	**Mar**	**Apr**	**May**	**Jun**	**Jul**	**Aug**	**Sep**	**Oct**	**Nov**	**Dec**	**Jan**	**Feb**		
Rotavirus	9	3	4	4	2	3	1	0	6	9	14	49	< 0.001	***
Rotavirus –Cut-off*	7	3	4	3	1	2	1	0	5	5	11	38	< 0.001	***
Norovirus GII	6	0	1	2	6	2	0	0	4	13	12	15	0.004	**
Norovirus GII –Cut-off	4	0	1	1	3	0	0	0	2	5	5	8	0.093	-
Shigella/EIEC	1	0	3	2	9	3	2	2	1	7	3	8	0.134	-
Shigella/EIEC –Cut-off	0	0	1	1	7	2	1	1	0	3	2	3	0.026	*
Campylobacter	1	3	2	2	5	5	0	5	2	5	4	6	0.207	-
Campylobacter –Cut-off	1	2	1	1	1	1	0	4	0	3	1	3	0.421	-
EAEC (*aggR*)	11	4	3	6	12	7	8	8	7	10	8	19	0.031	*
EAEC (*aggR*) –Cut-off	1	1	1	1	2	0	0	1	0	1	1	2	0.355	-
EAEC (*astA*)	18	9	7	6	20	14	11	16	18	27	25	50	0.270	-
EAEC (*astA*) –Cut-off	0	0	1	1	0	0	0	0	1	3	3	1	0.112	-
Aeromonas	7	4	4	4	4	5	1	9	6	4	8	10	0.100	-
Aeromonas –Cut-off	1	1	1	0	1	0	0	2	2	0	1	0	0.391	-
Sapovirus	0	0	0	1	5	2	0	0	2	2	3	0	0.138	-
Sapovirus –Cut-off	0	0	0	1	2	1	0	0	2	1	2	0	0.822	-
Astrovirus	0	1	0	0	0	1	0	0	0	5	4	2	0.100	-
Astrovirus –Cut-off	0	0	0	0	0	1	0	0	0	4	1	2	0.193	-
Salmonella	5	1	0	0	5	2	1	9	2	2	3	13	0.092	-
Salmonella –Cut-off	0	0	0	0	1	0	0	1	1	0	0	4	0.515	-
Plesiomonas	4	2	1	3	5	0	2	5	3	3	3	2	0.045	*
Plesiomonas –Cut-off	0	0	0	0	3	0	0	0	2	1	0	0	0.299	-
ETEC (*elt*)	2	1	0	0	5	4	2	0	3	7	4	7	0.154	-
ETEC (*elt*) –Cut-off	0	1	0	0	3	0	0	0	0	0	0	2	0.414	-

*, Pathogen positives under cut-off values (see Table 1).

Table S4. Basic information of the 370 diarrheal and 370 non-diarrheal control subjects

Characteristics	Diarrhea (%)	Control (%)	OR	*P*-value
No. of subject	370 (100)	370 (100)		
**Age group in years**				
< 5	241 (65.1)	232 (62.7)	ref	-
5-15	38 (10.3)	46 (12.4)	0.80 (0.5-1.27)	0.337
16-59	53 (14.3)	56 (15.1)	0.91 (0.6-1.38)	0.662
≥ 60	38 (10.3)	36 (9.7)	1.02 (0.62-1.66)	0.949
**Sex**				
Male	224 (60.5)	189 (51.1)	1.44 (1.08-1.94)	0.014
Female	146 (39.5)	181 (48.9)	ref	-
**Three periods (season)**				
Mar-Jun (hot)	77 (20.8)	108 (29.2)	-	-
Mar	29 (37.7)	60 (55.6)	0.71 (0.4-1.29)	0.264
Apr	18 (23.4)	20 (18.5)	1.33 (0.63-2.81)	0.456
May	12 (15.6)	12 (11.1)	1.48 (0.6-3.61)	0.393
Jun	18 (23.4)	16 (14.8)	1.66 (0.76-3.63)	0.203
Jul-Oct (rainy)	96 (25.9)	97 (26.2)	-	-
Jul	32 (33.3)	21 (21.6)	2.25 (1.15-4.43)	0.019
Aug	22 (22.9)	19 (19.6)	1.71 (0.82-3.55)	0.15
Sep	15 (15.6)	32 (33.0)	0.69 (0.33-1.44)	0.323
Oct	27 (28.1)	25 (25.8)	1.60 (0.82-3.12)	0.173
Nov-Feb (cool and dry)	197 (53.2)	165 (44.6)	-	-
Nov	27 (13.7)	26 (15.8)	1.53 (0.79-2.99)	0.208
Dec	38 (19.3)	24 (14.5)	2.34 (1.23-4.45)	0.01
Jan	44 (22.3)	65 (39.4)	ref	-
Feb	88 (44.7)	50 (30.3)	2.60 (1.54-4.38)	0
**Disease profile**				
Frequency of defecation				
≤ 5 times	176 (50.3)	-	-	-
6-9 times	79 (22.6)	-	-	-
≥10 times	115 (31.1)	-	-	-
Stool condition				
Semi solid	9 (2.4)	-	-	-
Watery	189 (51.1)	-	-	-
Liquid mucus	131 (35.4)	-	-	-
Mucus	24 (6.5)	-	-	-
Bloody mucus	17 (4.6)	-	-	-
Fecal WBC (abnormal)	16 (13.2)	-	-	-
Fecal RBC (presence)	17 (19.3)	-	-	-
**Systemic syndrome**				
Abdominal pain (Yes)	91 (25.1)	-	-	-
Vomiting (Yes)	216 (59.8)	-	-	-
Nausea (Yes)	85 (23.5)	-	-	-
Fever (Yes)	276 (76.5)	-	-	-

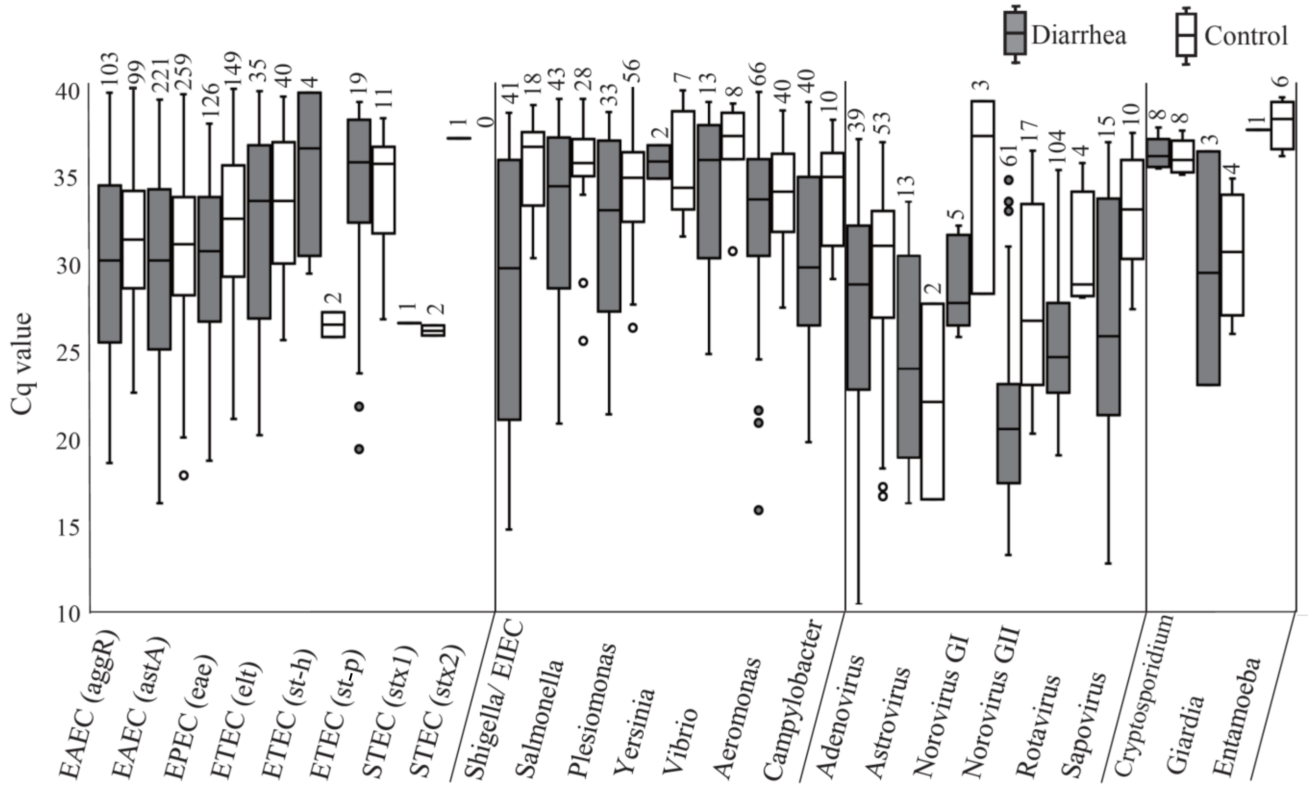

Figure S1. Box and whisker plot showing quantitation cycle (Cq) values of qPCR-positive samples for 24 target genes among 370 diarrheal and 370 non-diarrheal control subjects. The bottom and top of each box represent 25th and 75th percentiles of the data, respectively. The median value is displayed inside the box by the line. The maximum and minimum values are displayed with vertical lines connecting the points to the center box, along with the outliers represented herein indicated by closed and open circles. Numbers indicate the number of positive specimens.

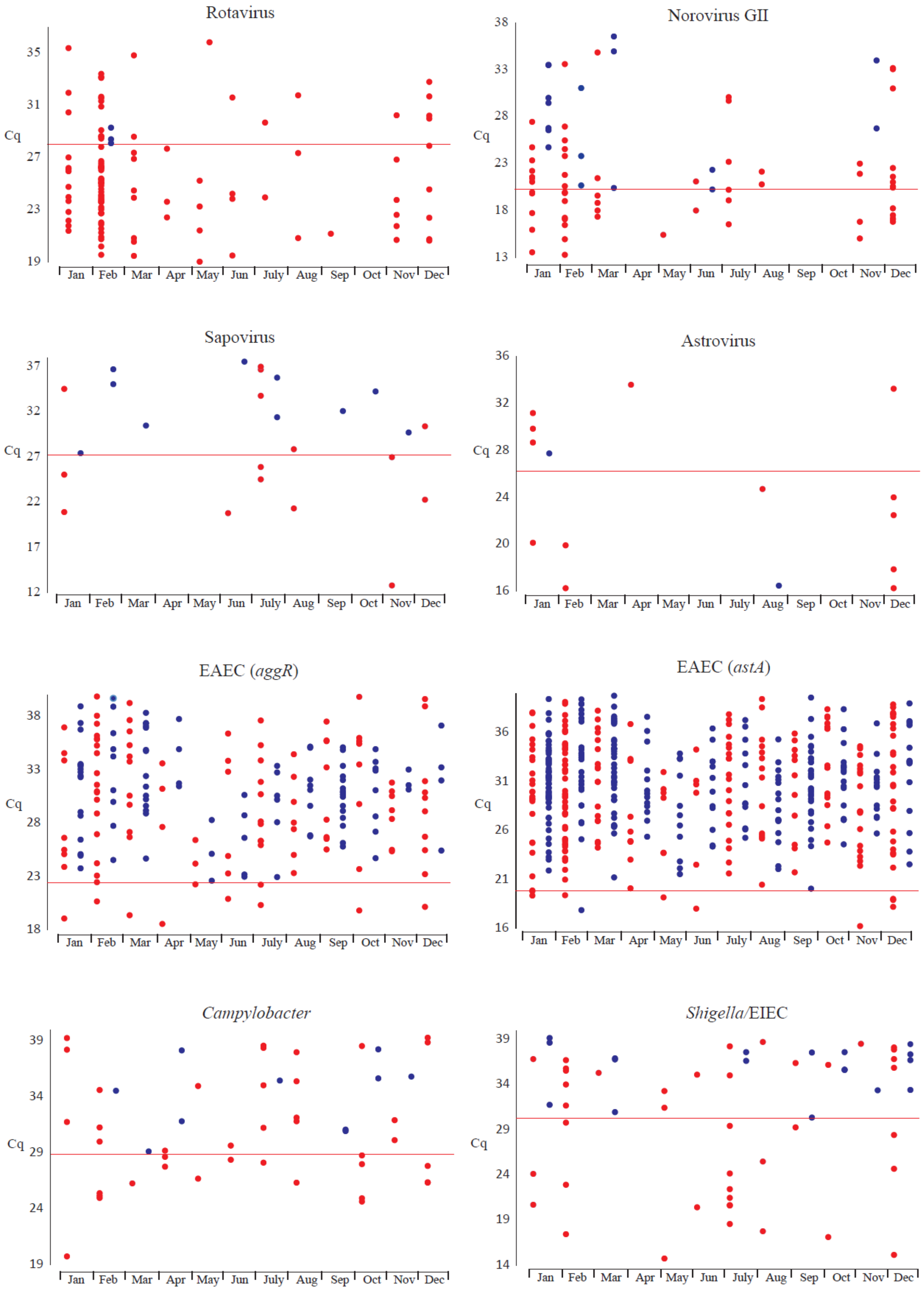

Rotavirus
Cq
35
31
27
23
19
Norovirus GII
38
33
28
23
18
13
Sapovirus
37
32
27
22
17
12
Astrovirus
36
32
28
24
20
16
EAEC (*aggR*)
38
33
28
23
18
EAEC (*astA*)
36
31
26
21
16
Campylobacter
39
34
29
24
19
Shigella/EIEC
39
34
29
24
19
14
Jan
Feb
Mar
Apr
May
Jun
July
Aug
Sep
Oct
Nov
Dec

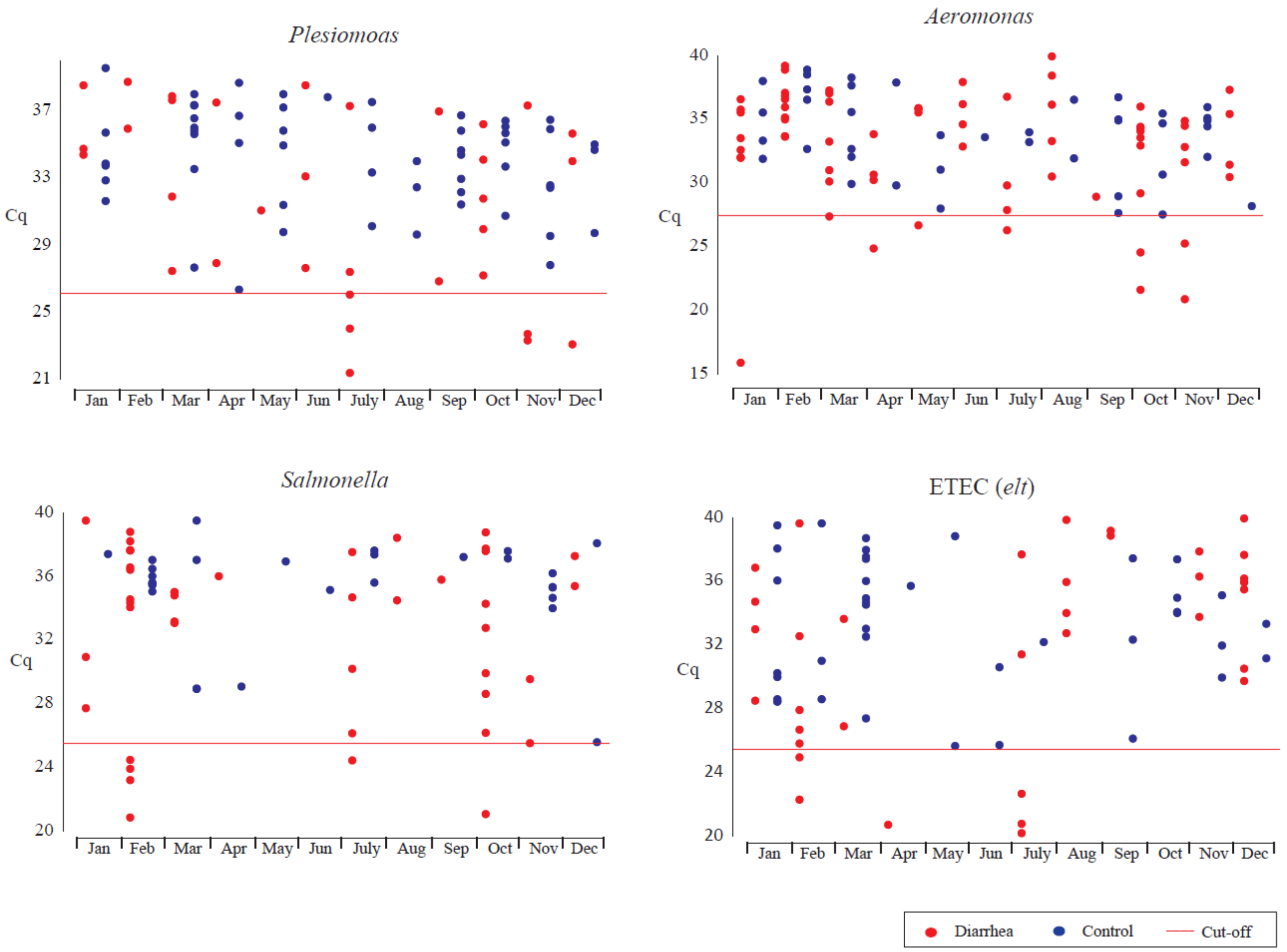

Figure S2. Detection of enteropathogens by month based on qPCR assays and distribution of Cq-values. The blue dots represent Cq values from the non-diarrheal control subjects and the red dots represent Cq values from the diarrheal patients. The red line indicates the cut-off value shown in Table 1.

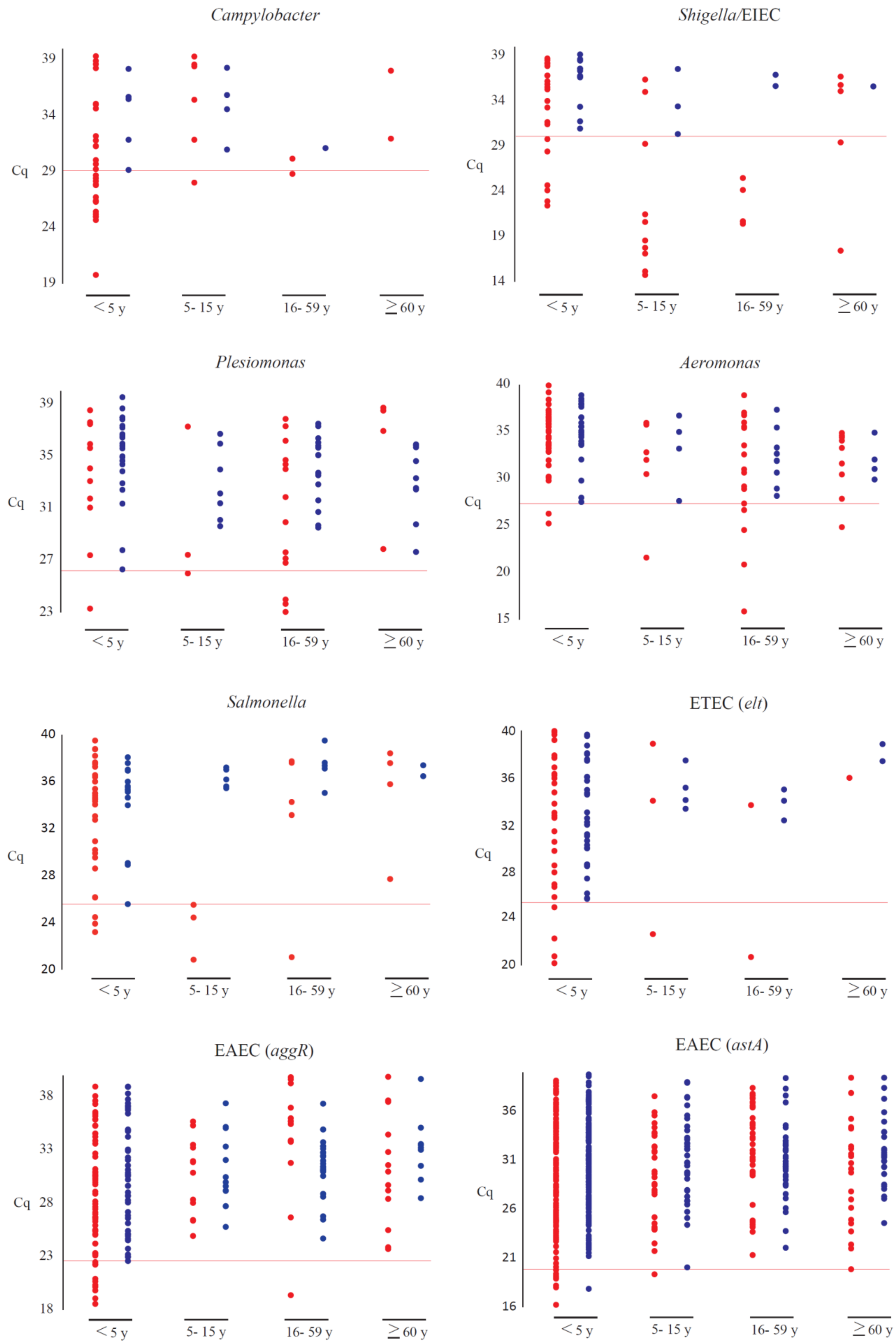

Campylobacter
Shigella/EIEC
Plesiomonas
Aeromonas
Salmonella
ETEC (*elt*)
EAEC (*aggR*)
EAEC (*astA*)
Cq
< 5 y
5- 15 y
16- 59 y
≥ 60 y

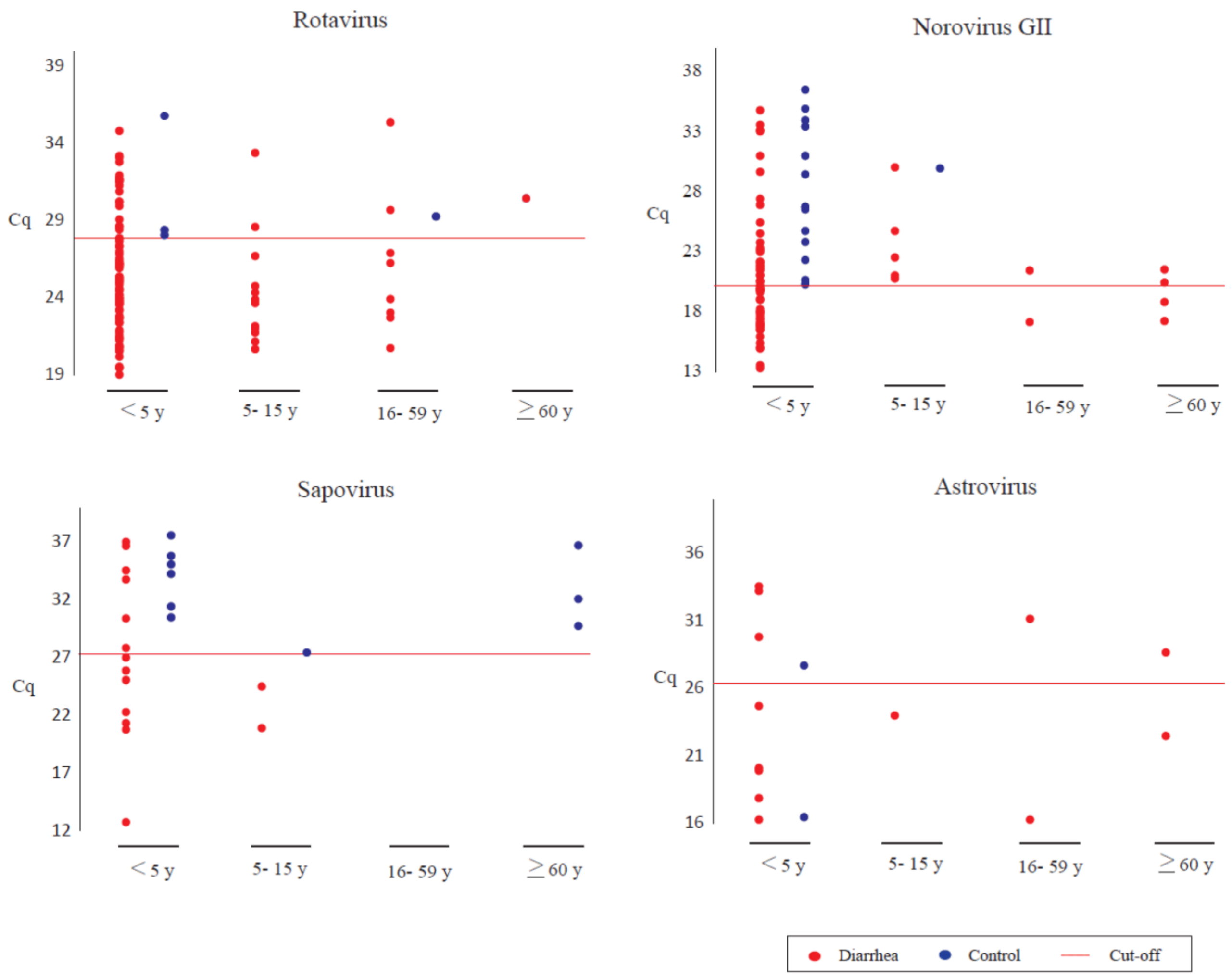

Figure S3. Detection of enteropathogens by age group using qPCR assays and distribution of Cq-values. The blue dots represent Cq values from the non-diarrheal control subjects and the red dots represent Cq values from the diarrheal patients. The red line indicates the cut-off value shown in Table 1.

Supplementary methods

We reported the patient baseline characteristics and disease profile with proportions, stratified by the groups. The relationships between disease profile and pathogens detected/not detected in the cases were presented as ORs and *P*-values were determined using the Fisher's exact test, as shown in Table 1. Similarly, we also reported the relationships between disease profile and Cq-value (positive/negative).
